# Role of bifidobacteria in the hydrolysis of chlorogenic acid

**DOI:** 10.1002/mbo3.219

**Published:** 2014-12-16

**Authors:** Stefano Raimondi, Andrew Anighoro, Andrea Quartieri, Alberto Amaretti, Francisco A Tomás-Barberán, Giulio Rastelli, Maddalena Rossi

**Affiliations:** 1 Department of Life Sciences, University of Modena and Reggio EmiliaItaly; 2 Department of Food Science and Technology, CEBAS-CSICMurcia, Spain

**Keywords:** Bifidobacteria, *Bifidobacterium animalis*, caffeic acid, chlorogenic acid, feruloyl esterase, hydroxycinnamic acids, probiotics

## Abstract

This study aimed to explore the capability of potentially probiotic bifidobacteria to hydrolyze chlorogenic acid into caffeic acid (CA), and to recognize the enzymes involved in this reaction. *Bifidobacterium* strains belonging to eight species occurring in the human gut were screened. The hydrolysis seemed peculiar of *Bifidobacterium animalis*, whereas the other species failed to release CA. Intracellular feruloyl esterase activity capable of hydrolyzing chlorogenic acid was detected only in *B. animalis*. In silico research among bifidobacteria esterases identified Balat_0669 as the cytosolic enzyme likely responsible of CA release in *B. animalis*. Comparative modeling of Balat_0669 and molecular docking studies support its role in chlorogenic acid hydrolysis. Expression, purification, and functional characterization of Balat_0669 in *Escherichia coli* were obtained as further validation. A possible role of *B. animalis* in the activation of hydroxycinnamic acids was demonstrated and new perspectives were opened in the development of new probiotics, specifically selected for the enhanced bioconversion of phytochemicals into bioactive compounds.

## Introduction

Edible plants provide the human with different non-nutritional phytochemicals which can exert a beneficial role in health. A major group of bioactive phytochemicals is represented by polyphenols, which include diverse classes of compounds, such as phenolic acids, flavonoids, lignans, and stilbenes (Manach et al. 2004). Among phenolic acids, hydroxycinnamic acids (HCA) are a major group of polyphenols exhibiting antioxidant, nitrite-scavenging, and anti-carcinogenetic activities (El-Seedi et al. 2012). They are distributed in plant tissues and occur abundantly in many plant-derived foods and beverages (e.g., fruits, berries, seeds, cereals, leafy vegetables, coffee). HCA are rarely found in free form and generally occur as oligomers or as esters formed by condensation with hydroxyl acids, alcohols, and carbohydrates (Scalbert and Williamson 2000; Manach et al. 2004; El-Seedi et al. 2012). Caffeic acid (CA) is the most abundant HCA in berry, fruits, and coffee, where it is present in remarkably high concentration. Thus, it is one of the most abundant polyphenols in diet, the daily intake of which may reach 1 g in coffee drinkers (El-Seedi et al. 2012). CA is commonly ester bound to quinic acid (QA), in the form of chlorogenic acid (3-o-caffeoyl-quinic acid, C-QA). Likewise for other HCA, the beneficial effects of C-QA and CA have been widely explored, confirming their antioxidant and DNA–protective activities (Xu et al. 2012; Jang et al. 2014). Recent studies also demonstrated the anti-diabetic effect of CA, the enhancement of vascular health through a potent antihypertensive activity with low toxic manifestations, the antiproliferative and cytotoxic properties in a variety of cancer cell lines without displaying significant toxicity toward healthy cells (Gomes et al. 2003; Mubarak et al. 2012; Ong et al. 2013; Bhullar et al. 2014; Oboh et al. 2014). Likewise other polyphenols affecting the composition of the colonic microbiota, CA was also demonstrated to inhibit harmful intestinal bacteria such as opportunistic pathogens (Lou et al. 2011; Duda-Chodak 2012).

Like most HCA esters, the major part of C-QA is not absorbed in the small intestine and reaches the colon (Azuma et al. 2000; Stalmach et al. 2010). Here it encounters the commensal microbiota, which is capable to perform synergistic metabolic transformations affecting its fate and the biological activity. Interindividual differences in excretion profiles of HCA metabolites have been observed, indicating that the composition of the microbiota may dictate the fate of dietary HCA, affecting their bioavailability and activity (Olthof et al. 2003; Rechner et al. 2004). Microbial metabolism of C-QA includes the hydrolysis to QA and CA, making free CA available for absorption. Besides, free CA may be further transformed by colonic bacteria into a variety of bioactive metabolites, such as m-coumaric acid and hydroxylated derivatives of phenylpropionic and benzoic acids (Gonthier et al. 2003, 2006; Ogawa et al. 2011; Tomàs-Barberàn et al. 2014). A more complex picture of C-QA fate has been recently depicted, since the colonic microbiota can hydrogenate or dehydroxylate C-QA before hydrolyzing the ester bond, or even breaking the QA moiety (Tomàs-Barberàn et al. 2014). Bacterial biotransformation generally converge on 3-(3-hydroxyphenyl)-propanoic acid, even though the order of the reactions may differ among subjects, resulting from differences in microbiota composition.

The colonic microbiota is composed largely of anaerobic bacteria, with cell numbers exceeding 10^11^ per gram of intestinal content. In adults, it is dominated by bacteria belonging to the phyla *Firmicutes*, *Bacteroidetes*, and *Actinobacteria* (Arumugam et al. 2011). *Firmicutes* are by far the most abundant and assorted group and include the Clostridia and Bacilli classes. *Actinobacteria* constitute a sub-dominant proportion (up to 8%) of bacterial population, mostly composed of bacteria belonging to the genus *Bifidobacterium* (Arumugam et al. 2011). Bifidobacteria are one of the most important health-promoting groups of the colonic microbiota and are used as probiotics (Rossi and Amaretti 2010). They exert beneficial effects through different mechanisms, such as immunostimulation, anticarcinogenic activity, pathogen growth inhibition, vitamin and amino acid production, reduction of the conversion of primary bile salts to secondary bile salts, bioconversion of a number of dietary compounds into bioactive healthy molecules (Rossi and Amaretti 2010).

This study investigated the role of bifidobacteria in C-QA metabolism. Couteau et al. (2001) isolated six intestinal strains capable of hydrolyzing HCA, among which a sole *Bifidobacterium* isolate. To our knowledge, a comprehensive screening exploring the capability of bifidobacteria to transform HCA has never been accomplished. We wanted to fill this gap by investigating the capability of these health-promoting bacteria to transform C-QA, and characterizing the enzyme involved in the ester bound hydrolysis.

## Material and Methods

### Chemicals, bacterial strains, and culture conditions

All chemicals were purchased from Sigma-Aldrich (Steinheim, Germany) unless otherwise stated. Stock solutions of 75 mmol/L C-QA and CA acid were prepared dissolving each substance in dimethyl sulfoxide. Thirty-two strains of *Bifidobacterium* (Table1), taken from our collection of human isolates or obtained from ATCC American Type Culture Collection, Manassas, VA, USA, were subcultured at 37°C in *Lactobacillus* MRS broth (BD Difco, Sparks, MD) containing 0.5 g/L L-cysteine HCl (hereinafter called MRS) in an anaerobic cabinet under a N_2_ 85%, CO_2_ 10%, H_2_ 5% atmosphere.

**Table 1:** Yield of C-QA hydrolysis after 24 h cultivation of bifidobacteria in presence of 0.5 mmol/L C-QA, and ability to grow in MRS supplemented with 0.5, 2, and 10 mmol/L CA or C-QA

Strain	Yield^1^ C-QA → CA	Growth with CA (mmol/L)			Growth with C-QA (mmol/L)		
	%	0.5	2	10	0.5	2	10
*B. animalis* subsp*. animalis* ATCC 27536	28 ± 1	+	+	+	+	+	+
*B. animalis* subsp. *animalis* WC 0409	28 ± 3	+	+	+	+	+	+
*B. animalis* subsp*. animalis* WC 0410	28 ± 2	+	+	+	+	+	+
*B. animalis* subsp*. animalis* WC 0411	7 ± 1	+	+	−	+	+	+
*B. animalis* subsp. *lactis* WC 0412	17 ± 1	+	+	−	+	+	+
*B. animalis* subsp. *lactis* WC 0413	41 ± 3	+	+	+	+	+	+
*B. animalis* subsp. *lactis* WC 0414	34 ± 3	+	+	+	+	+	+
*B. animalis* subsp. *lactis* WC 0432	50 ± 2	+	+	+	+	+	+
*B. animalis* subsp*. lactis* WC 0471	27 ± 2	+	+	+	+	+	+
*B. bifidum* WC 0415	0	+	+	−	+	+	+
*B. bifidum* WC 0417	0	+	+	−	+	+	+/−
*B. bifidum* WC 0418	0	+	+	−	+	+	+
*B. breve* WC 0420	0	+	+	−	+	+	+
*B. breve* WC 0421	0	+	+	−	+	+	+
*B. breve* WC 0422	0	+	+	−	+	+	+
*B. breve* WC 0423	0	+	+	−	+	+	+
*B. breve* WC 0424	0	+	+	−	+	+	+
*B. breve* WC 0473	0	+	+	+/−	+	+	+
*B. catenulatum* ATCC 27539	0	+	+	+	+	+	+
*B. catenulatum* WC 0458	0	+	+	−	+	+	+
*B. catenulatum* WC 0467	0	+	−	−	+	+	−
*B. longum* subsp*. infantis* ATCC 15697	0	+	+	+	+	+	+
*B. longum* subsp. *infantis* WC 0433	0	+	+	−	+	+	−
*B. longum* subsp. *infantis* WC 0434	0	+	+	−	+	+	−
*B. longum* subsp. *longum* WC 0436	0	+	+	−	+	−	−
*B. longum* subsp. *longum* WC 0438	0	+	+	−	+	+	−
*B. longum* subsp. *longum* WC 0439	0	+	+	−	+	+	+
*B. longum* subsp. *longum* WC 0440	0	+	+	−	+	+	+
*B. longum* subsp. *longum* WC 0443	5 ± 2	+	+	−	+	+	+
*B. pseudocatenulatum* WC 0400	0	+	+	−	+	+	−
*B. pseudocatenulatum* WC 0401	0	+	+	−	+	+	+
*B. pseudocatenulatum* WC 0402	0	+	−	−	+	+	−
*B. pseudocatenulatum* WC 0403	0	+	+	−	+	+	+
*B. pseudocatenulatum* WC 0407	0	+	−	−	+	+	−
*B. pseudocatenulatum* WC 0408	0	+	−	−	+	+	−

1 Data are means ± SD of three separate experiments.

To evaluate the resistance of bifidobacteria to C-QA and CA, 16 h MRS cultures were inoculated (10% v/v) into 5 mL of MRS containing 0, 0.5, 2, and 10 mmol/L C-QA or CA. The cultures were incubated anaerobically for 24 h, then the turbidity at 600 nm (OD_600_) was determined. Strains were considered as inhibited if final OD_600_ was the 25% or less than in MRS control cultures. To evaluate C-QA transformation, 16 h MRS cultures were inoculated (10% v/v) into 5 mL of MRS containing 500 *μ*mol/L C-QA, and incubated at 37°C in anaerobiosis for 48 h. As controls, 5 mL of culture without C-QA and 5 mL of noninoculated medium containing 500 *μ*mol/L C-QA were carried out. Bioreactor batch processes were performed with *Bifidobacterium animalis* subsp. *lactis* WC 0432 in 500 mL bioreactors (Sixfors V3.01; Infors, Bottmingen, Swiss), containing 250 mL of MRS supplemented with 500 *μ*mol/L C-QA. The culture was kept at 37°C under gentle agitation; anaerobic conditions were maintained by keeping the medium under a stream of CO_2_; if necessary for the specific experiment, a pH controller delivered 4 mol/L NaOH to maintain the pH at 5.5. Samples were collected periodically to monitor turbidity and C-QA hydrolysis.

### Bioconversion of C-QA with supernatants, resting cells, and cell extracts

Supernatants, resting cells, and cell extracts were prepared from MRS cultures and were utilized for bioconversion experiments to investigate the location of C-QA hydrolase activity. Culture samples were centrifuged (6000*g* for 10 min at 4°C), then the supernatant was filtered at 0.22 *μ*m and used for C-QA bioconversion. The pellet was washed twice and suspended with 50 mmol/L phosphate buffered saline (50 mmol/L, pH 6.5), then adjusted to 3.0 units of OD_600_. The suspension was divided into aliquots that were directly utilized as resting cells biocatalyst for C-QA bioconversion or were mechanically disrupted. To prepare the extract, the cellular suspension was given one stroke at 40.0 kPsi in One Shot Cell Disrupter (Constant Systems), then it was centrifuged to remove solids (13,000*g* for 15 min at 4°C) and filtered through 0.22 *μ*m filter. Supernatants, resting cells, and cell-free extracts were incubated at 37°C for 24 h in presence of 500 *μ*mol/L C-QA. In negative control reactions, CQA was incubated with the buffer or with supernatants, resting cells, or cell-free extracts that were inactivated at 100°C for 10 min. To examine in depth the enzyme location in *B. animalis* subsp. *lactis* WC 0432, MRS batch cultures (constant pH = 5.5) were sampled at 4, 20, 24, and 36 h. C-QA hydrolysis was assayed in the supernatants, resting cells, and cell extracts from different growth phases. To quantify the extent of cell lysis, fructose-6-phosphate phosphoketolase (F-6-PPK) was assayed spectrophotometrically at 540 nm in both cell-free extracts and dialyzed supernatants (Tannock 1999). Cell viability was determined by means of a fluorescence-based assay kit for microscopy (Live/Dead BacLight; Life Technologies, Thermo Fisher Scientific, Waltham, MA, USA).

### In silico search of Bifidobacterium esterases

The sequence of cinnamoyl esterase Lj0536 from *Lactobacillus johnsonii* (Lai et al. 2011) was utilized as *tblastn* query in the search for homologs in the sequenced genomes of *B. animalis* (accession numbers CP001213.1, CP001515.1, CP001606.1, CP001853.1, CP001892.1, CP002567.1, CP002915.1, CP003039.2, CP003497.1, CP003498.1, CP003941.1, and CP004053.1) (Altschul et al. 1990).

The genes annotated as “esterase” in the genome of *B. animalis* subsp. *lactis* DSM 10140 (CP001606.1) were translated and used as *tblastn* queries to search the homologous within the nucleotide sequences of *B. bifidum*, *B. breve*, *B. catenulatum*, *B. longum* subsp. *longum*, and *B. longum* subsp. *infantis*, and *B. pseudocatenulatum*, including the genomes of *B. bifidum* (NC_017999.1, NC_014638.1, and NC_014616.1), *B. breve* (CP006711.1, CP006715.1, CP002743.1, CP006712.1, and CP006713.1), *B. longum* (NC_004307.2 and NC_010816.1), and *B. longum* subsp. *infantis* (NC_015052.1 and NC_011593.1). Signal P 4.0 and Secretome P 2.0 servers were used to predict the presence of signal peptides driving secretion and protein location (Bendtsen et al. 2005; Petersen et al. 2011).

### Homology modeling and molecular docking

The sequence alignments were performed with Protein Blast against the Protein Data Bank (PDB) with default parameters as set in the web interface (Altschul et al. 1990; Berman et al. 2000). The query sequence of Balat_0669 was obtained from the NCBI protein database (NCBI reference sequence: YP_002969671.1). Alignments were repeated for confirmation with ClustalW2 (Larkin et al. 2007; Goujon et al. 2010).

The homology modeling of Balat_0669 was performed with Modeller 9.11 (Šali and Blundell 1993). The cinnamoyl esterase and p-nitrobenzyl esterase X-ray crystal structures were downloaded from the PDB (PDB ID: 3PF8 and 1QE3). For each protein, 500 models were built, DOPE score was calculated and used to rank the generated models. The last five C-terminal residues of Balat_0669 could not be modeled because the corresponding residues in Lj0536 were missing in the 3PF8 crystal structure. This region is about 24 Å from the esterase active site, hence the deletion of such a limited of residues is assumed to have no effect on substrate binding. The refinement of the homology model was performed with Amber modules (Case et al. 2008). AM1-BCC atomic partial charges were calculated for the ligands with Antechamber and the minimization was performed with Sander (Goujon et al. 2010). The parameters were set as follows: a distance-dependent dielectric constant, a 12 Å cutoff for nonbonded interactions, and 500 steps of conjugate gradient energy minimization.

The structure of Balat_0669 homology model was prepared by adding hydrogen atoms with Amber's module Leap (Case et al. 2008). Gasteiger partial atomic charges were added to the protein with MGLTools AutoDock Tools 1.5.6 (Sanner 1999; Morris et al. 2009). The initial conformations of the ligands were generated with the program LigPrep (version 2.5; Schrödinger, LLC, New York, NY, 2011). Gasteiger charges were assigned to the ligands with MGLTools 1.5.6. Docking was performed with Autodock 4.2.3 (Morris et al. 2009) and parameters were set as follows: 50 runs of genetic algorithm, 2.500.000 maximum energy evaluations, 150 individuals in a population, and a root mean squared deviation (RMSD) tolerance of 2 Å (Jain 2008). Grids were centered on the binding site identified by CA in 3S2Z for Balat_0669. The grid dimensions were 47 × 40 × 40 grid points, with a spacing of 0.375 Å. Refinement of docking complexes was performed via energy minimization with Sander (Case et al. 2008), with parameters identical to those used to refine the homology model (Rastelli 2013).

### Primers design and cloning of Balat_0669

Genomic DNA of *B. animalis* subsp. *lactis* WC 0432 was used as template after purification using the kit DNA Blood & Tissue (Qiagen, Hilden, Germany), following the manufacturer protocol for Gram-positive bacteria. The primer pair 0669-F (5′-CCATATGACGACGAGCACAC-3′) and 0669-R (5′-AAGCTTCACGCCACCTCATG-3′) was designed according to the gene sequence of the homolog of Balat_0669 in *B. animalis* subsp. *lactis* DSM 10140, to obtain the whole amplification of the open reading frame (*orf*). The restriction sites *Nde*I and *Hin*dIII were introduced in the forward and in the reverse primer, respectively. In order to be easily purified from *Escherichia coli*, the recombinant protein Balat_0669 was also cloned and expressed fused to the C-terminal 6 × His tag carried in the expression vector pET-21b(+). The primers 0669-F and 0669-R2 (5′- CTC GAG CGC CAC CTC ATG ATG-3′), were used to amplify the Balat_0669 gene without the stop codon, introducing the restriction sites *Nde*I and *Xho*I, respectively.

The PCR products (789 bp and 796) were cloned into the vector pTZ57R using the InsTAclone PCR cloning kit (Thermo Fisher Scientific, Waltham, MA). *E. coli* DH5*α* was transformed with the ligation mixtures using the TransformAid Bacterial Transformation Kit (Thermo Fisher Scientific). The genes were excised from the recombinant plasmid pTZ-669 and pTZ-669His with *Nde*I-*Hin*dIII and *Nde*I-*Xho*I digestion, respectively. The fragments were ligated into the expression vector pET-21b(+), resulting in the recombinant plasmids pET-669 and pET-669His.

### Functional expression of the esterase Balat_0669 in *E. coli*

The expression was assessed on *E. coli* BL21 (DE3) harboring pET-699. The culture was incubated at 30°C until OD_600_ = 1.0, then protein expression was induced with 1 mmol/L isopropyl-*β*-d-1-thiogalactopyranoside (IPTG) for 0.5, 1, 3, 5, 8, and 24 h at 30°C. Whole cells proteins were separated with sodium dodecyl sulphate polyacrylamide gel electrophoresis (SDS-PAGE) using the buffer system of Laemmli (1970) and 10% acrylamide gels. Gel images were acquired by the GS800 calibrated densitometer (Bio-Rad) and analyzed by the image analysis software “Quantity One” (Bio-Rad, Redmond, WA, USA).

Gene function was confirmed performing *E. coli* resting cell biotransformations. 0.1 L of Luria Bertani broth containing ampicillin 100 g/L (LB-Amp) were inoculated with an overnight culture in order to obtain an OD_600_ = 0.2. The culture was incubated at 30°C (180 rpm) until an OD_600_ = 1, then protein expression was induced with 1 mmol/L IPTG for 5 h. The cells were washed three times with PBS pH 6.5 and resuspended to a final OD_600_ = 2.0. Ten milliliters of resting cells were supplemented with 1 mmol/L C-QA and incubated at 30°C (180 rpm) for 24 h, monitoring C-QA conversion and CA production after 1, 2, 4, 8, 16, and 24 h. As control, a parallel biotransformation was performed with the biomass of *E. coli* BL21 (DE3) harboring the empty vector pET-21b.

### Purification and enzymatic assay of the esterase Balat_0669-(His)_6_

The 6 × His tagged protein was obtained from *E. coli* BL21 (DE3) harboring pET-699His. The strain was inoculated in 1 L of LB-Amp and grown at 30°C until OD_600_ = 1.0. The expression was induced with 1 mmol/L IPTG, and the culture was incubated at 25°C for 16 h. The biomass was collected by centrifugation and resuspended (200 g/L) in binding buffer (20 mmol/L sodium phosphate, 500 mmol/L NaCl, 20 mmol/L imidazole, pH 7.4) containing a protease inhibitor cocktail (P8465; Sigma-Aldrich). Cells were lysed by sonication in ice (15 burst of 30 sec followed by intervals of 30 sec for cooling) and debrides were removed by 15 min centrifugation at 50000*g* and 4°C. Clear supernatant was applied onto a AKTA Prime Plus system, equipped with three HisTrap FF Crude 5 mL columns (GE Healthcare, Buckinghamshire, UK) serially combined. The stationary phase was initially washed with 50 mL of binding buffer (3 mL/min) and the release of unbounded proteins was monitored measuring the absorbance at 280 nm in the eluate. Bounded proteins were eluted by a gradient of 20–500 mmol/L imidazole, the fractions containing recombinant proteins were pooled, and dialyzed against 20 mmol/L Tris-HCl buffer containing 0.1 mol/L NaCl, pH 6.5. Protein purification was monitored by SDS-PAGE and protein yield was measured by spectrophotometric quantification using the Bradford assay (500-0006; Bio-Rad).

The esterase activity of the purified Balat0669-(His)_6_, was assayed at 30°C, in 20 mmol/L Tris-HCl pH6.5, containing 1 mmol/L C-QA The reaction was monitored by HPLC analysis. The activity was expressed as micromoles of C-QA hydrolyzed per minute per milligram of enzyme.

### Analysis of C-QA and CA

C-QA and CA were analyzed by HPLC, with a device (HPLC 1100; Agilent Technologies, Waldbronn, Germany) equipped with a ZORBAX Eclipse XDB-C18 (Agilent) column and ultraviolet-visible diode array detector. A volume of 10 *μ*L of sample was injected. Elution was performed at room temperature with 0.7 mL/min of a mixture of 0.1% (v/v) acetic acid in water (phase A) and 0.1% (v/v) acetic acid in acetonitrile, (phase B). The following gradient of phase B was applied: 0–6 min, linear from 10% to 15%; 6–14 min, linear to 100%; 14–21 min, isocratic on 100%; 21–24 min, linear to 10%; 24–30 min, isocratic on 10%. The analytes C-QA and CA were identified at 280 nm and were quantified by external standard method. Linearity was demonstrated from 25 to 1000 *μ*mol/L (*R*^2^ > 0.995). The limits of detection (LOD) of C-QA and CA were 0.30 and 0.35 *μ*mol/L, respectively. LOD was calculated as 3·(Sy/x)/b, where Sy/x represents the residual SD and b is the slope of the linear calibration.

### Statistical analysis

All values are means of three separate experiments. Differences in means were analyzed using analysis of variance (ANOVA), followed by Tukey post hoc comparisons (SPSS version 20; IBM, Armonk, NY). Differences were considered statistically significant for *P *<* *0.05.

## Results

### Resistance to C-QA and CA

Thirty-two strains belonging to eight *Bifidobacterium* species occurring in the human gut were screened for their ability to grow in the presence of different concentrations of C-QA or CA (Table1). All the strains grew abundantly with both 500 *μ*mol/L C-QA or CA, without significant differences in final turbidity, compared with MRS (*P *>* *0.05, data not shown). Bifidobacteria grew generally well also in presence of 2 mmol/L C-QA or CA, with few exceptions within *B. catenulatum*/*B. pseudocatenulatum* group and *B. longum* species. A greater number of strains belonging to *B. longum* and *B. catenulatum*/*B. pseudocatenulatum* became uncapable of growth in presence of 10 mmol/L C-QA, while *B. animalis*, *B. bifidum*, and *B. breve* remained uninhibited . The majority of the strains were inhibited by 10 mmol/L CA, with exceptions mostly belonging to *B. animalis* subsp. *lactis* and *B. animalis* subsp. *animalis*.

### Hydrolysis of C-QA by bifidobacteria

The ability of bifidobacteria to hydrolyze C-QA into CA and QA was investigated. C-QA hydrolysis did not occur in control noninoculated media. All the strains grew well in C-QA-supplemented medium and gave biomass yields similar (*P *>* *0.05) to the ones obtained in control MRS cultures (data not shown).

Only 10 out of 32 strains hydrolyzed C-QA and released CA. Among these, all the 9 strains of *B. animalis* subsp. *animalis* and *B. animalis* subsp. *lactis* (7 to 50% CA) were present along with the strain *B. longum* subsp. *infantis* WC0443 (5% CA) as the sole representative of the other species. The most effective strain was *B. animalis* subsp. *lactis* WC 0432, which accomplished a 50% hydrolysis. All the strains of *B. bifidum*, *B. breve*, *B. catenulatum*, *B. pseudocatenulatum*, and most of *B. longum* were incapable to transform C-QA at any extent. CA and residual C-QA always accounted for more than 98% of initial C-QA, while metabolites originating from reductive reactions were never found.

C-QA biotransformation experiments were carried out also with the supernatants and the cell-free extracts obtained from stationary MRS cultures. Both the cell-free extracts and the supernatants prepared from the strains that were able to hydrolyze C-QA during growth were active as well. On the contrary, the cell-free extracts and the supernatants prepared from strains other than *B. animalis* and *B. longum* subsp. *infantis* WC 0443 were always inactive in C-QA hydrolysis.

### C-QA hydrolysis by *B. animalis* subsp. lactis WC 0432

**Figure 1:**
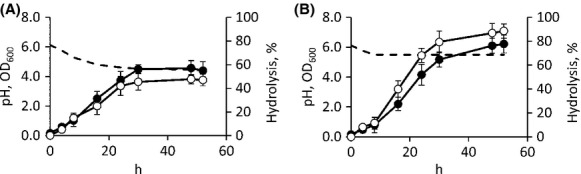
Time-course of C-QA hydrolysis in batch cultures of *Bifidobacterium animalis* subsp. lactis WC 0432 with the pH left uncontrolled (A) or prevented to decrease below 5.5 (B). The strain was cultured in MRS broth supplemented with 500 *μ*mol/L C-QA. Symbols: ●, turbidity, ○, C-QA hydrolysis (%), dashed line, pH. Data are means of three separate experiments, bars represent SD, for pH, SD always <0.1.

To investigate the kinetics of C-QA hydrolysis by *B. animalis* subsp. *lactis* WC 0432, the strain performing the highest conversion, bioreactor batch cultures were performed. The strain was cultured in MRS broth containing 500 *μ*mol/L C-QA with or without pH control. In the absence of control, the pH gradually decreased to 4.5 due to the accumulation of lactic and acetic acids, and growth ended after 30 h, yielding an OD_600_ of 4.6. C-QA hydrolysis accompanied the growth and reached a yield of 40% (Fig.1A). Higher biomass production and C-QA hydrolysis (*P *<* *0.05) were both achieved if pH was prevented from decreasing below 5.5 (Fig.1B). In this condition, the culture grew up to OD_600_ = 6.2, while up to 88.6% C-QA was hydrolyzed, mostly during the growth phase (68.2% in the first 24 h).

The location of the enzyme responsible for C-QA hydrolysis in *B. animalis* subsp. *lactis* WC 0432 was investigated through biotransformation experiments. 500 *μ*mol/L C-QA were incubated for 24 h in presence of cell-free extracts, resting cells, and supernatants that were prepared from MRS cultures (constant pH = 5.5) at exponential, decelerating, early stationary, and late stationary phases (i.e., after 4, 20, 24, and 48 h of growth, respectively). All the cell-free extracts effectively hydrolyzed C-QA, yielding similar conversions of 87 ± 4 to 91 ± 4%, regardless of the growth phase (*P *>* *0.05). On the contrary, all the resting cells performed the hydrolysis at a lower extent, giving conversions of 6 ± 3 to 11 ± 2% regardless of the growth phase (*P *>* *0.05). Supernatants performed C-QA hydrolysis with increasing efficiency (*P *<* *0.05), resulting in 7, 21, 39, and 67% of conversion (SD always <4%) at exponential, decelerating, early stationary, and late stationary phases, respectively. Supernatants were inactivated if pH was corrected to 4.5.

To determine whether the hydrolytic activity of supernatants could have originated from secretion or from cell lysis, an intracellular enzyme involved in sugar metabolism, F-6-PPK, was quantified in cell-free extracts and supernatants. Increasing F-6-PPK activity was found in supernatants throughout the growth, indicating the release of cytoplasmic enzymes: at the decelerating phase, F-6-PPK and C-QA esterase activities presented intracellular/extracellular ratios of 9.3 and 13.5, respectively. Consistently, fluorescence-based cell viability assay confirmed that, during the process, the percentage of damaged bacteria increased from 5% at the exponential phase to 23% at the late stationary phase.

### In silico search of Bifidobacterium esterases

All the sequenced genomes from both *B. animalis* subsp. *lactis* and *B. animalis* subsp. *animalis* bear a homologue of Lj0536, a cinnamoyl esterase from *L. johnsonii* able to hydrolyze C-QA (query coverage = 90%; identity = 42%). The sequences retrieved from the genomes of *B. animalis* are identical and, likewise Lj0536, do not present any homologue in other *Bifidobacterium* species. In *B. animalis* subsp. *lactis* DSM 10140 the homologue of Lj0536 is Balat_0669 and is annotated as “esterase.” The genome of this strain presents nine other sequences annotated as “esterase”: Balat_0183, Balat_0519, Balat_0593, Balat_0859, Balat_0899, Balat_1050, Balat_1264, Balat_1547, Balat_1604. The search for their homologues within each of the species that were unable to hydrolyze C-QA (*B. bifidum*, *B. breve*, *B. longum* subsp. *longum*, and *B. longum* subsp. *infantis*, and *B. catenulatum* and *B. pseudocatenulatum*) was fruitful, yielding putative homologues in at least three other species (Table S1).

Balat_0669 lacks any signal peptide for Sec or TAT secretion pathways and is likely intracellular, based on Secretome P 2.0 and Signal P 4.0 predictions.

### Homology models and docking analysis

**Figure 2:**
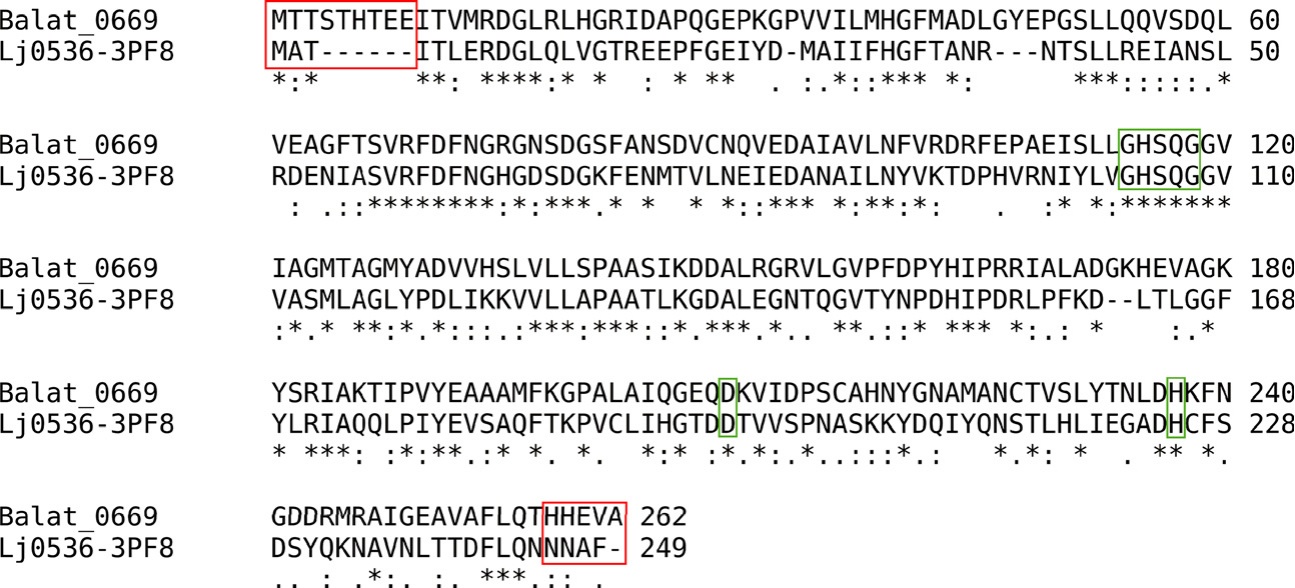
Sequence alignment between Balat 0669 and Lj0536. The alignment of the catalytic triad and serine protease motif is highlighted in green. The N-term and C-term residues excluded from the homology model building are highlighted in red.

Having hypothesized Balat_0669 being an esterase able to catalyze the hydrolysis of C-QA into CA and QA, it was evaluated whether the substrate binds the active site and makes interactions compatible with catalysis. Since the X-ray crystal structure of Balat_0669 has not been solved, the *Lactobacillus johnsonii* cinnamoyl esterase Lj0536 (Lj0536) (PDB ID: 3PF8) was identified in the Protein Data Bank as the top scoring homolog and as a reasonable template for homology modeling. It was also reported that the esterase 3FP8 catalyzes the hydrolysis of C-QA (Lai et al. 2011). The alignment between the two sequences resulted in 42% identity with a 95% query coverage (Fig.2). The Lj0536 catalytic triad formed by Ser106, His225, and Asp197 corresponded to residues Ser116, His227, and Asp209 of Balat_0669. Furthermore, the serine esterase conserved motif (Gly-X-Ser-X-Gly) formed by Gly104-His105-Ser106-Gln107-Gly108 of Lj0536 was correctly aligned with Gly114-His115-Ser116-Gln117-Gly118 of Balat_0669 (Fig.2). Five hundred models of the protein were built, among which a final representative with high score and high query/template similarity of active site residue conformations was chosen. The PDB also contains the crystal structure of the inactive S106A mutant of Lj0536 in complex with CA (PDB ID: 3S2Z) (Lai et al. 2011). This structure was used to refine the model since it contains a bound ligand and it provides a suitable active site architecture for docking studies. Structural refinement of the selected Balat_0669 model was carried out with energy minimization after superimposing Balat_0669 and 3S2Z structures, and manually placing CA taken from the superimposed 3S2Z structure into the Balat_0669 structure. Two water molecules (waters 1149 and 1240 in 3S2Z) that form a network of hydrogen bonds between the protein and the ligand were included in the model. Energy minimization of the residues in a range of 4 Å from the ligand was carried out.

**Figure 3:**
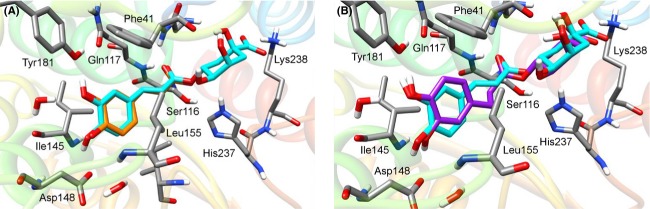
Refined docking complexes. (A) Docking orientation of C-QA (cyan) in the binding site of Balat_0669. X-ray crystallographic orientation of CA (orange) as in 3S2Z structure of Lj0536 is also shown for a comparison. (B) Superposition of the docking orientations of C-QA (cyan) and DHPP-QA (purple) in Balat_0669.

As a validation of the docking procedure, CA was initially redocked in the Balat_0669 refined model. The orientation of CA was strikingly similar to that observed in the 3S2Z crystal structure, with a RMSD of only 0.4 Å, therefore the docking method as well as the Balat_0669 structure resulted appropriate for our purposes. C-QA was docked using the same parameters and the resulting complex was refined through energy minimization. The CA moiety of C-QA binds the protein very similarly to CA of 3S2Z, as shown in Figure3A. Remarkably, the ester bond of C-QA is at a short distance and almost perpendicular to the Ser116 oxygen, and therefore is ideally positioned to undergo catalysis, confirming that the predicted binding mode is consistent with the expected hydrolysis mechanism. In addition, the QA moiety forms additional favorable interactions with Balat_0669: its two hydroxyls make hydrogen bonds with the backbone carbonyl of Phe41, and the carboxylate establishes a strong salt bridge with Lys238. The free energy of binding estimated by AutoDock is −8.8 Kcal/mol.

Dihydrochlorogenic acid (DHPP-QA) was suggested to be hydrolyzed to dihydrocaffeic acid (DHPPA) by esterase activity (Tomàs-Barberàn et al. 2014) and, thus, it may be another putative substrate for Balat_0669. To assess its ability to bind the esterase, this molecule has been docked with the same parameters used for C-QA. The resulting complex displayed a binding mode comparable to the one predicted for Balat_0669/C-QA, as shown in Figure3B. The hydrogen bond network between the ligand and the binding site residues was conserved and the ester bond was again positioned favorably for catalysis. The estimated free energy of binding for this complex was −9.8 Kcal/mol, that is, close to the one observed for C-QA.

In conclusion, the binding modes predicted by AutoDock for C-QA and DHPP-QA in complex with Balat_0669 are consistent with a putative hydrolytic esterase activity, the ester bond being in close contact with the catalytic triad and a number of favorable electrostatic and hydrophobic interactions being formed.

### Expression of Balat _0669 in *E. coli* and functional characterization

**Figure 4:**
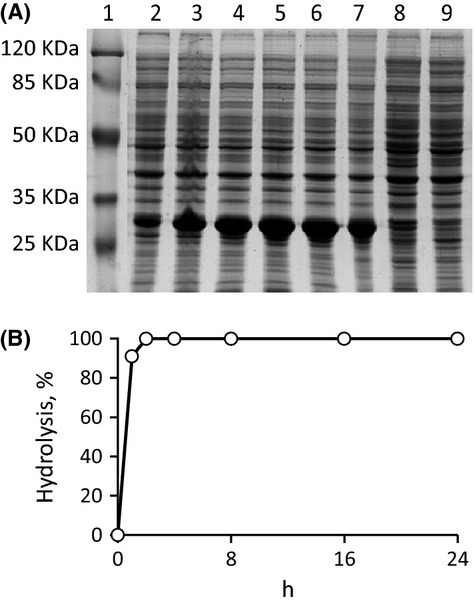
Expression of the esterase Balat_0669 in *Escherichia coli* BL21 (DE3) holding pET-669 vector. (A) SDS-PAGE analysis of whole cells proteins (10% acrylamide in Laemmli buffer system, approx. 40 *μ*g per lane): lane 1, prestained protein molecular weight marker (SM0441, Fermentas); lanes 2 to 7, samples after 0.5, 1, 3, 5, 8, 24 h of isopropyl-*β*-d-1-thiogalactopyranoside (IPTG) induction; lane 8, uninduced control; lane 9, *E. coli* BL21 (DE3) holding pET21b empty vector. (B) Time-course of C-QA hydrolysis by resting cells of *E. coli* expressing the esterase Balat_0669. Values are means, *n* = 3, SD always <4%.

In order to determine the suitability of *E. coli* BL21 (DE3) for cloning and expressing Balat_0669, the absence of homologous genes on its genome was ascertained. Furthermore, the expression host, harboring the empty vector pET-21b(+), was incapable to perform any C-QA transformation (data not shown). A primer pair designed on the Balat_0669 coding sequence of *B. animalis* subsp. *lactis* DSM 10140 was used to clone the *orf* of *B. animalis* subsp. *lactis* WC 0432. The coding sequence from *B. animalis* subsp. *lactis* WC 0432 consisted of 789 bp, and was identical to that of *B. animalis* subsp. *lactis* DSM 10140. *E. coli* BL21 was transformed with the expression vector pET-669, bearing the gene for Balat_0669. SDS-PAGE revealed that expression occurred at high level in IPTG-induced recombinant cells, reaching the highest value 5 h after the induction, when recombinant Balat_0669 accounted for the 37% of *E. coli* proteins (Fig.4). Resting cells of induced *E. coli* expressing Balat_0669 were incubated with C-QA 1 mmol/L and its concentration was checked hourly via HPLC analysis. Conversion of C-QA into CA was 91% after 1 h, and was complete after 2 h of incubation.

**Figure 5:**
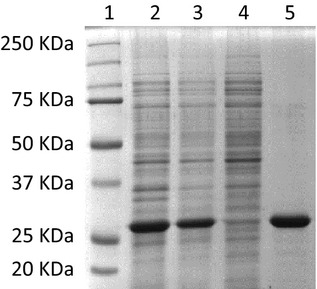
Purification of the esterase Balat_0669-(His)_6_ expressed in *Escherichia coli* BL21 (DE3) holding pET-669His vector. (A) SDS-PAGE analysis: lane 1, prestained protein molecular weight marker (161-0374; Bio-Rad); lane 2, crude lysate; lane 3, cell-free extract; lane 4, unbound fractions; lane 5, pooled purified fractions.

Balat_0669 was also tagged at the C terminus with a (His)_6_ sequence, expressed in *E. coli* BL21 (DE3) and purified by immobilized metal affinity chromatography (IMAC). From 1 L of LB culture approximately 100 mg of protein were purified close to homogeneity as ascertained by SDS-PAGE electrophoresis (Fig.5). The C-QA esterase activity was evaluated by incubating the recombinant protein with 1 mmol/L C-QA, monitoring substrate conversion, and CA release. The specific activity in the purified fraction was 2.5 U/mg, approximately four times higher than in the crude lysate (0.47 U/mg).

## Discussion

Biotransformation of plant precursors in health-promoting molecules has recently been claimed as a mechanism involved in the positive-health effects of bifidobacteria. The production of a cinnamoyl esterase capable of releasing CA was suggested by previous investigations: a *Bifidobacterium lactis* strain produced a cell-bound cinnamoyl esterase active against C-QA and ethyl ferulic acid (Couteau et al. 2001), and a *B. bifidum* strain was able to utilize feruloyl oligosaccharides as carbon sources removing feruloyl groups (Yuan et al. 2007). Therefore, a possible role of bifidobacteria in the release of caffeic and ferulic acids in the colon should be considered as a possible health effect, and the production of hydroxycinnamoyl-esterases by bifidobacteria deserves a deeper investigation. This study aimed to explore the role of bifidobacteria in C-QA hydrolysis and CA release, and to recognize the enzymes involved in this reaction.

Although a wide range of health-promoting activities of dietary HCA has been described, their effect on the modulation of the gut ecology has been poorly investigated (Etxeberria et al. 2013). As far as C-QA and CA are concerned, it is known that CA exerts inhibitory activity against certain pathogenic bacteria, with MIC values that may be quite low (e.g., 0.056 mmol/L against *Streptococcus pneumoniae* and *Shigella dysenteriae*) (Lou et al. 2011). Then, it is possible that these phytochemicals contribute to modulation of colonic microbiota inhibiting in a different manner the diverse microbial groups. The vast majority of bifidobacteria, including those that do not participate in C-QA hydrolysis, were resistant to 2 mmol/L C-QA. This concentration approximates the highest that may occur in the colon, considering 1 g/day C-QA dietary uptake, 30% absorption in the small intestine, and 1 kg of colonic content. Furthermore, it should be considered that C-QA reaching intact the colon is promptly transformed by the gut microbiota and its concentration rapidly decreases (Selma et al. 2009). As a whole, bifidobacteria are not negatively affected by physiological amounts of C-QA or CA. However, some opportunistic pathogens may be affected by polyphenols (Lou et al. 2011; Duda-Chodak 2012), indicating that inhibition of harmful microbial groups may be another mechanism responsible of the positive-health effects of these phytochemicals.

The screening carried out in order to determine the capability of different *Bifidobacterium* species of human origin to hydrolyze C-QA revealed that all the strains belonging to both the *B. animalis* subspecies (*B. animalis* subsp. *animalis* and *B. animalis* subsp. *lactis*) performed this transformation, in agreement with previous data (Couteau et al. 2001). Bifidobacteria belonging to the species *B. bifidum*, *B. breve*, *B. catenulatum*, *B. pseudocatenulatum*, and to the subspecies *B. longum* subsp. *infantis* and *B. longum* subsp. *longum* failed to perform this transformation, with a sole exception (*B. longum* subsp. *longum* WC 0443). To determine whether the inability of most of the tested species to hydrolyze C-QA could be ascribed to the absence of an esterase able to hydrolyze this substrate, or to the lack of membrane transporters involved in making the substrate available to cytosolic enzymes, a set of transformations carried out with supernatants or cell-free extracts obtained from early stationary cultures was performed. As a result, the strains unable to hydrolyze C-QA did not show detectable enzyme activity.

The available genome sequences of bifidobacteria were analyzed to identify the genetic determinants that make *B. animalis* able to hydrolyze C-QA. Among all the sequences annotated as esterases in bifidobacteria genomes, only Balat_0669 is peculiar of *B. animalis*, being absent in the species unable to hydrolyze C-QA. Due to its high similarity, Balat_0669 is also the only bifidobacteria esterase that could be considered a homolog of Lj0536, a cinnamoyl esterase from *L. johnsonii* able to hydrolyze C-QA, and was then hypothesized to be responsible for the same reaction in *B. animalis*. Balat _0669 does not bear a signal sequence for Sec or TAT secretion pathways, in agreement with the evidence that *B. animalis* subsp. *lactis* WC 0432 harbors an intracellular C-QA-esterase.

For the first time, the structure of Balat_0669 has been predicted through comparative modeling and the binding of C-QA has been investigated with molecular docking. Analysis of the resulting C-QA–esterase complexes showed that C-QA binds the active site with favorable electrostatic and hydrophobic interactions and places the ester bond in a position prone to catalysis by the catalytic serine. Cloning and expressing the homolog of Balat _0669 from *B. animalis* subsp. *lactis* WC 0432 in *E. coli* confirmed that it is an enzyme capable of converting C-QA.

In a previous study, *B. animalis* subsp. *lactis* WC 0432, was added to colonic microbiota cultures supplemented with 1.5 mmol/L C-QA in order to improve the flux toward CA (Tomàs-Barberàn et al. 2014). Compared with control microbiota cultures, the presence of *B. animalis* subsp. *lactis* WC 0432 did not foster the release of CA, because C-QA hydrogenation yielding DHPP-QA proceeded faster than ester hydrolysis. Nonetheless, it is plausible that *B. animalis* subsp. *lactis* WC 0432 hydrolyzed DHPP-QA and accelerated the release of DHPPA, because a greater accumulation of DHPPA occurred in presence of the probiotic strain. Docking of DHPP-QA into the Balat_0669 structure displayed a binding mode comparable to the one predicted for Balat_0669/C-QA. Thus, it is conceivable that Balat_0669 esterase of *B. animalis* subsp. *lactis* WC 0432 did not contribute to C-QA hydrolysis due to the competition by other reactions, including reduction to DHPP-QA, while it may have played a role in DHPPA release. In addition to the direct activity of probiotics on dietary phytochemicals, their activity on metabolites coming from microbiota metabolism is worth investigating.

The strategy exploited in the present study highlights the necessity to associate genomic data to a functional approach in the study of colonic microbiota activities. In fact, the analysis of genome sequences for predictable metabolic reactions cannot be sufficient for estimating whether *Bifidobacterium* strains or other colonic bacteria can transform dietary molecules or xenobiotics. The combined approach used in this investigation can be taken as a model for a deep insight into the study of the function of colonic bacteria, and especially of probiotics.

## Conflict of Interest

None declared.

## Supporting Information

**Table S1.** Results of the search, by tblastn analysis, for homologs of the 10 annotated esterases of *B. animalis* subsp. *lactis* DSM 10140, in all other *Bifidobacterium* species that were unable to hydrolyze C-QA (*B. bifidum*, *B. breve*, *B. longum* subsp. *longum*, and *B. longum* subsp. *infantis*, and *B. catenulatum,* and *B. pseudocatenulatum*).
