# Supplementary material for: Role of bifidobacteria in the hydrolysis of chlorogenic acid
Source: Microbiologyopen. 2014 Dec 16;4(1):41–52. doi: 10.1002/mbo3.219 (PMC4335975; doi:10.1002/mbo3.219)
Supplement: Supplementary file 1 [file mbo30004-0041-sd1.pdf]

**Table S1**. tblastn analysis of ten esterases from *B. animalis* subsp. *lactis* DSM 10140 against other *Bifidobacterium* species. For each query, the top-scoring match from each species is reported.

| Query | Species | Max score | Query coverage | Max identity | Accession |
|---|---|---|---|---|---|
| Balat_0183 | *B. bifidum* | 249 | 98 | 42 | WP_003819218.1 |
| | *B. breve* | 238 | 94 | 43 | WP_003828717.1 |
| | *B. catenulatum* | 240 | 94 | 42 | WP_003834207.1 |
| | *B. longum* subsp. *infantis* | 241 | 94 | 44 | YP_004209323.1 |
| | *B. longum* subsp. *longum* | 241 | 94 | 44 | YP_005586887.1 |
| | *B. pseudocatenulatum* | 241 | 94 | 42 | WP_004220784.1 |
| Balat_0519 | *B. bifidum* | 40 | 33 | 28 | YP_003970982.1 |
| | *B. breve* | 56 | 32 | 35 | YP_005583272.1 |
| | *B. catenulatum* | 446 | 91 | 61 | WP_003836035.1 |
| | *B. longum* subsp. *infantis* | 56 | 32 | 35 | YP_004209468.1 |
| | *B. longum* subsp. *longum* | 412 | 91 | 57 | YP_003661869.1 |
| | *B. pseudocatenulatum* | 445 | 91 | 61 | WP_004220040.1 |
| Balat_0593 | *B. bifidum* | 432 | 95 | 49 | WP_003821849.1 |
| | *B. breve* | 399 | 94 | 47 | WP_016462045.1 |
| | *B. catenulatum* | 338 | 99 | 39 | WP_003835320.1 |
| | *B. longum* subsp. *infantis* | 419 | 94 | 48 | YP_004207988.1 |
| | *B. longum* subsp. *longum* | 424 | 95 | 48 | EIJ24166.1 |
| | *B. pseudocatenulatum* | 162 | 39 | 43 | WP_004221494.1 |
| Balat_0669 | *B. bifidum* | 31 | 23 | 34 | WP_003821651.1 |
| | *B. breve* | 30 | 43 | 25 | WP_019727705.1 |
| | *B. catenulatum* | 29 | 22 | 33 | WP_003836509.1 |
| | *B. longum* subsp. *infantis* | 30 | 23 | 31 | EEQ56135.1 |
| | *B. longum* subsp. *longum* | 30 | 23 | 31 | YP_003999647.1 |
| | *B. pseudocatenulatum* | 29 | 43 | 25 | EEG70556.1 |
| Balat_0859 | *B. bifidum* | 164 | 96 | 48 | YP_006394573.1 |
| | *B. breve* | 184 | 91 | 45 | YP_005582750.1 |
| | *B. catenulatum* | 167 | 90 | 48 | WP_003834200.1 |
| | *B. longum* subsp. *infantis* | 183 | 92 | 48 | YP_004208853.1 |
| | *B. longum* subsp. *longum* | 186 | 92 | 48 | EIJ22703.1 |
| | *B. pseudocatenulatum* | 171 | 95 | 47 | WP_004220797.1 |
| Balat_0899 | *B. bifidum* | 278 | 96 | 42 | WP_003812916.1 |
| | *B. breve* | 32 | 67 | 26 | WP_021649292.1 |
| | *B. catenulatum* | 286 | 95 | 46 | WP_003835048.1 |
| | *B. longum* subsp. *infantis* | 281 | 89 | 47 | YP_002323033.1 |
| | *B. longum* subsp. *longum* | 276 | 89 | 45 | EIJ24322.1 |
| | *B. pseudocatenulatum* | 279 | 95 | 46 | EEG70556.1 |
| Balat_1050 | *B. bifidum* | 38 | 30 | 25 | YP_003970982.1 |
| | *B. breve* | 180 | 90 | 34 | WP_003831575.1 |
| | *B. catenulatum* | 333 | 92 | 51 | WP_003833915.1 |
| | *B. longum* subsp. *infantis* | 139 | 86 | 31 | YP_002323832.1 |
| | *B. longum* subsp. *longum* | 243 | 90 | 41 | YP_005586728.1 |
| | *B. pseudocatenulatum* | 298 | 92 | 51 | WP_004223447.1 |

| Query | Species | Max score | Query coverage | Max identity | Accession |
|---|---|---|---|---|---|
| Balat_1264 | *B. bifidum* | 419 | 97 | 70 | YP_003970608.1 |
| | *B. breve* | 398 | 96 | 66 | WP_003832590.1 |
| | *B. catenulatum* | 435 | 99 | 62 | WP_003834207.1 |
| | *B. longum* subsp. *infantis* | 400 | 96 | 67 | YP_002322248.1 |
| | *B. longum* subsp. *longum* | 404 | 98 | 68 | YP_003661660.1 |
| | *B. pseudocatenulatum* | 436 | 99 | 71 | WP_004220784.1 |
| Balat_1547 | *B. bifidum* | 337 | 99 | 59 | WP_021647798.1 |
| | *B. breve* | 45 | 67 | 26 | WP_016463038.1 |
| | *B. catenulatum* | 30 | 37 | 27 | WP_003833915.1 |
| | *B. longum* subsp. *infantis* | 374 | 99 | 66 | YP_002321557.1 |
| | *B. longum* subsp. *longum* | 339 | 99 | 61 | EIJ23907.1 |
| | *B. pseudocatenulatum* | 383 | 100 | 68 | WP_004223450.1 |
| Balat_1604 | *B. bifidum* | 315 | 77 | 63 | YP_003939530.1 |
| | *B. breve* | 310 | 81 | 61 | YP_005583710.1 |
| | *B. catenulatum* | 340 | 77 | 67 | WP_003835361.1 |
| | *B. longum* subsp. *infantis* | 299 | 80 | 60 | YP_004209856.1 |
| | *B. longum* subsp. *longum* | 299 | 80 | 60 | EEI80347.1 |
| | *B. pseudocatenulatum* | 340 | 77 | 68 | WP_004223872.1 |
